# The Quebec Diabetes Empowerment Group Program: Program Description and Considerations Regarding Feasibility and Acceptability of Implementation in Primary Health Care Settings

**DOI:** 10.3389/fnut.2021.621238

**Published:** 2021-03-12

**Authors:** Fanny Hersson-Edery, Jennifer Reoch, Justin Gagnon

**Affiliations:** ^1^Department of Family Medicine, McGill University, Montreal, QC, Canada; ^2^Santé Kildare, Côte Saint-Luc, QC, Canada

**Keywords:** diabetes mellitus, family practice, patient-centered care, qualitative research & analysis, self-care, diabetes knowledge, self-efficacy

## Abstract

**Introduction:** Diabetes is a highly prevalent chronic disease that frequently coexists with other medical conditions and implies a high burden for patients and the healthcare system. Clinicians currently are challenged to provide effective interventions that are both multidisciplinary and empower patient self-care. The Diabetes Empowerment Group Program (DEGP) was developed with the aims of fostering patient engagement in diabetes self-care through the lens of empowerment and to support the empowerment of patients with diabetes by providing multidisciplinary group-based care. This research's objectives were to: (1) develop a comprehensive description of the DEGP for potential adopters, and (2) explore the factors influencing the feasibility and acceptability of implementing it in other healthcare settings in Montreal.

**Methods:** A qualitative descriptive study was conducted, following a participatory approach. Data were obtained from: (1) semi-structured interviews with 14 patients who participated in the pilot program; (2) from semi-structured group interviews with patient partners, healthcare professionals, and other stakeholders from 4 Montreal family medicine groups, and (3) discussions among the participatory research team during various knowledge translation activities. Inductive content analysis of the data was performed.

**Results:** The DEGP identified seven key elements: medical visit, continuity of care, group-based dynamics, multi-disciplinarity, clinician facilitation, patient-centered agenda, and a theoretical framework of empowerment. The content and organization of the group visits were conceived to address each of these four domains. The empowerment framework comprises four domains of self-care: emotional (attitude), cognitive (knowledge), behavioral (skills), and relational (relatedness). Factors impacting the feasibility and acceptability of implementing the DEGP in other primary care settings were identified.

**Discussion:** The DEGP fits within the discourse around the need for more patient-centered programs for people living with diabetes, following a more comprehensive empowerment model. This research could facilitate the development or adaptation of similar programs in other settings.

## Introduction

Diabetes Mellitus is among the most prevalent chronic diseases and represents a major burden for health care systems, primary health care services, and patients. It is estimated that 3.4 million people in Canada (9.3% of the population) live with diabetes (1). Furthermore, 22% of the population is estimated to be prediabetic (2). Given that almost 80% of diabetes care occurs at the primary care level, adequate diabetes management in the primary care setting is essential (3).

Diabetes management and lifestyle changes recommended in the Diabetes Canada 2018 Clinical Practice Guidelines may be difficult for many patients. Furthermore, the complexity of managing diabetes can be overwhelming for patients and primary care providers. In Canada, medical care for people with diabetes generally comprises individual physician visits, with referral to specialists and allied professionals as needed. In accordance with current initiatives to foster evidence-based and patient-centered medicine, Diabetes Canada recommends a multidisciplinary approach that includes physicians, nurse practitioners, nurses, pharmacists, dieticians, and psychological health workers to support individuals managing their diabetes (3). Evidence also suggests that diabetes management is improved when patients are empowered and engaged in self-care (4, 5). Therefore, two key elements can be identified in this expert recommendation: (1) the provision of care by a multidisciplinary clinical group and (2) increase a person's control over his/her medical condition.

In response to these diabetes care needs, The Diabetes Empowerment Group Program (DEGP) was developed and piloted at Santé Kildare, a Family Medicine Group (FMG) in Montreal, Canada. The pilot aimed to assess the feasibility of the DEGP and refine it using participant feedback. Three cohorts of 6–8 patients (21 in total) living with diabetes (type 1 or type 2) or pre-diabetes participated in 6 sessions over the course of 3 months. It was conceived as a collaborative multidisciplinary patient-centered program that aims to stimulate patient empowerment and enable the development of a community of individuals who support one another.

Effective knowledge translation strategies are needed to support its implementation throughout Montreal. Knowledge translation is reinforced by using a robust, scientific process to develop a detailed description of the intervention that speaks to potential adopters and identification of the factors that impact its implementation. Two of these factors include the feasibility and acceptability of implementing the intervention at other sites and by other stakeholders.

This research's objectives were to: (1) develop a comprehensive description of the DEGP for potential adopters, and (2) explore the factors influencing the feasibility and acceptability of implementing it in other healthcare settings in Montreal. The knowledge generated by this research is expected to support the implementation and adaptation of similar patient-centered diabetes empowerment programs in other healthcare settings.

## Methods

### Study Design

A qualitative descriptive study design (6) with a participatory research approach (7) was used for both study objectives. A participatory approach was used whereby knowledge users' inclusion in the research process supported the generation of relevant and meaningful knowledge, and the translation of this knowledge into practice. Collaboration between researchers and non-academic stakeholders supports the generation of more meaningful knowledge and more effective knowledge translation (7, 8). The research team consisted of: three researchers from the Department of Family Medicine of McGill University, a family physician (DEGP physician), a nurse practitioner (DEGP nurse), and three patient partners. The three patient partners had been participants in the DEGP pilot (9). All members of the research team collaborated throughout the entire research process, including: the definition of objectives, data collection, data analysis, and dissemination of results (10).

### Data Collection

Data collection took place between 2015 and 2017. Data were obtained from three main sources. The description of the DEGP was informed by: semi-structured interviews were conducted with patients who had participated in the 2015 DEGP pilot; and data generated by the research team's ongoing reflection on the program during the development of knowledge translation tools. Perceptions regarding the feasibility and acceptability were identified using group interviews with patient partners and healthcare providers.

#### Description of the DEGP

Regarding semi-structured interviews, all 21 patients who had participated in the pilot were approached to participate in interviews. Fourteen of these patients agreed to participate. The interview guide was developed inductively by the research team. Questions posed to patients emphasized their perceptions of the program and recommendations for improvement. Interviews were recorded and transcribed.

The research team's reflection also informed the program's description throughout the development of knowledge translation tools such as logic models, program implementation guides, manuscript preparation, patient recruitment brochure creation, and scientific conference presentation. The DEGP physician, the DEGP nurse, and the three patient partners had been involved in the pilot. As the research was conducted, more detailed reflections on the program came to light. These reflections were documented in personal notes and meeting minutes.

#### Exploration of Feasibility and Acceptability

Factors influencing the feasibility and acceptability of implementing a similar or adapted group program were elicited from 3 group interviews. The group interview discussions included patient partners, healthcare providers (physicians and nurses), allied health professionals (kinesiologist, dietician, pharmacist) and administrators (clinic managers and clinic coordinators, quality improvement coordinator) from 4 primary care family medicine clinics in Montreal (Santé Kildare, Herzl Family Practice Center, CLSC Cote-des-Neiges, CLSC Parc-Extension). One of three patient partners participated in each of the group interviews. The group interview guide was developed by the research team using a hybrid approach. The questions emphasized the feasibility and acceptability of implementing the DEGP in different settings. The Diffusion of Innovations framework informed the initial questions (11). The research team then added additional questions to address the research objectives and elements specific to the DEGP. The DEGP physician facilitated the group interviews, and two observers took detailed notes. The interviews were audio-recorded and transcribed.

### Analysis

Inductive thematic analysis (12) was performed on the patient interview transcripts and meeting notes to draw out key elements of the DEGP. A trained research assistant identified initial codes representing sub-themes. The research team generated additional sub-themes. The themes and sub-themes and were used in the construction of a description of the DEGP. This description led to the generation of a logic model to explain desired outcomes of interest to various stakeholders.

Considerations for implementation in other settings was coded using a hybrid approach (13) (deductive and inductive), using Greenhalgh et al.'s Diffusion of Innovations in Health Organizations framework (11) to establish broad themes and assign emergent sub-themes to these. The research assistant identified initial codes representing sub-themes. The sub-themes were discussed, validated, categorized, and interpreted by the research team. The research team discussed these themes and sub-themes in relation to Greenhalgh et al.'s Diffusion of Innovations in Health Organizations framework. This led to the description of acceptability and transferability of the DEGP in other Montreal settings presented below.

### Ethics

Ethics approval was obtained from the McGill University Faculty of Medicine Institutional Review Board. All research team members and all participants in the individual and group interviews provided written consent before participating.

## Results

### Description of the Diabetes Empowerment Group Program (DEGP)

The DEGP was conceived as a series of group medical visits with a fixed cohort of 6–8 patients. Meetings take place twice a month for the first 8 visits, then for one year thereafter (or longer if there is interest) meetings are held on a monthly basis. The group is facilitated by a family physician or nurse practitioner and another allied health professional. A broad theme is discussed at each visit (Table 1). The sessions, however, are open-ended and flexible in order to promote participation and pertinent discussion. The facilitator's role is key to help guide conversation and provide factual information as needed. The purpose of discussions is to share experiences, increase knowledge, teach skills, foster mutual support, and promote a greater sense of control over one's health.

**Table 1:** Examples of discussion topics by DEGP visit.

**Visit/Theme (2 h)**	**Discussion Topics**	**Possible Tools**
**Group visit 1 - Introduction to the program** Facilitators: Family Physician and Nurse	• Introductions • Structure of the program • Confidentiality • Empowerment construct • Personal experience living with diabetes • Beliefs and values related to diabetes	• Ice breakers • Empowerment framework illustration • Describe your diabetes path • Write down fears and challenges related to diabetes
**Group visit 2 - Understanding Diabetes** Facilitators: Family Physician and Nurse	• What is diabetes? • “Pathophysiology” of my disease • Symptoms of hyperglycemia and hypoglycemia • Checking my sugars	• Illustrations of organs and body systems • Glucometer demonstration • Tools for tracking sugars • Write down challenges related to hypo or hyper-glycemia
**Group visit 3 - Lifestyle habits (part 1):** **food and nutrition** Facilitators: Family Physician, Nurse and **Dietician**	What is safe to eat, what to avoid and why?	• Portion sizing • Balanced plate • Food labels • Field trip to grocery store • Online resources • Collective kitchen • Restaurant choices
**Group visit 4 - Medications** Facilitators: Family Physician, Nurse and **Pharmacist**	• How my medications work • Side effects • Medication compliance	• Resources • Illustrations and videos of medication action • My diabetes journal
**Group visit 5 - Foot care** Facilitators: Family Physician, Nurse and **Podiatrist**	• My feet and their importance • Foot health • What does a podiatrist do?	• Individual foot exam • Shoe exams • Resource to buy appropriate footwear
**Group visit 6 - Lifestyle habits (part 2):** **physical activity and exercise** Facilitators: Family Physician, Nurse and **Exercise specialist** or **physical trainer**	• Physical activity and exercise • Consequences of sedentary lifestyle • Making exercise a regular part of my day	• Podometer • Community resources • Demonstrating/teaching exercises • Exercise prescription • Online resources

Reflection and discussion of the DEGP contributed to the identification of seven key principles that characterize this intervention. These elements comprise: (1) medical visit, (2) continuity of care, (3) group-based dynamic, (4) multidisciplinary collaborative team, (5) facilitation by clinicians, (6) patient-centered agenda, and (7) based on an empowerment framework. Those interested in implementing this intervention should address each element and consider how they may be adapted according to their setting's resources and context. The following briefly describes how each principle was considered a key advantage of the intervention compared to standard diabetes medical care in the pilot program at Sante Kildare.

#### Medical Visit

Each of the DEGP meetings is considered a formal medical visit. In the pilot program, the DEGP involved up to 8 patients at once. An advantage of this program is that clinicians can spend as much time with the group as they would in individual visits, except the time spent with each patient is increased.

#### Continuity of Care

The DEGP involves a fixed cohort of patients and clinician facilitators. Compared to a drop-in approach, this program better ensures continuity with healthcare providers, facilitates a cumulative curriculum, and fosters social support networks among patients.

#### Group-Based Dynamic

Group visits have been recognized as a powerful tool for growth and change (14–16). They facilitate exposure to multiple perspectives, mutual support, encouragement, and feedback in a safe environment. Groups enhance self-management education and skills-building. Group visits also reinforce messages that are received in individual visits. Modeling, peer problem solving, and social support may also reduce perceived barriers to change in attitudes and behaviors.

#### Multidisciplinary Collaborative Team

Comprehensive care involves different health professionals working together in a mutually respectful manner, acknowledging the value of each other's discipline-specific skills, training, attributes, and contribution to diabetes care. During the pilot project, the multidisciplinary team consisted of a physician, nurse, pharmacist, nutritionist, podiatrist, and health coach. Other professionals may be invited based on the theme of the discussion.

#### Facilitation by Clinicians

The clinician facilitates the discussion, as opposed to lecturing. The facilitators draw on their experience using counseling skills, such as active listening and motivational interviewing, to encourage open and collaborative discussion. The facilitators ensure that every participant is engaged in discussion by asking open-ended questions, gently inviting less-talkative participants to contribute, and steering the conversation when one topic or one individual dominates the discussion.

#### Patient-Centered Agenda

Although each visit has a general theme, it is important that the information discussed be of interest and relevant to the participants (Table 1: Examples of discussion topics by DEGP visit). In order to maintain a patient-centered agenda, guest presenters and facilitators adjust the content of the discussion based on questions and concerns raised by participants. No formal, didactic presentation is given at each visit; however, the presenter may prepare topics of discussion or activities that may (or may not) be used depending on patient interest. This is often more enjoyable and requires preparation for the guest presenters.

#### Content Based on Empowerment Framework

The concept of empowerment has been proposed as a framework for engaging patients in self-care (17). The DEGP was developed around the theoretical framework of psychological empowerment, developed by Zimmerman (18) and adapted by Christens (19), comprising four components: attitude, knowledge, behavior, and relatedness.

*Attitude* includes the individuals' beliefs about themselves, including self-efficacy, perceived competence and control, and motivation. *Knowledge* refers to an individual's understanding of their community and includes critical awareness, understanding of causal factors, skills, and resource utilization. *Behavior* refers to the actions they take to effect change, such as: community involvement, skills development, organizational participation, and coping behaviors. Finally, *relatedness* refers to the psychological aspects of interpersonal interaction that underlie effective psychological empowerment. These include: collaborative competence, bridging social divisions, facilitating others' empowerment, mobilizing networks, and passing on a legacy.

### Additional Knowledge Translation Activities Supporting the Description of the DGEP

Understanding of the DEGP, in terms of its constituent elements and more nuanced features, was further refined throughout the knowledge translation (diffusion and dissemination) processes. These activities helped develop a more elaborate description of the intervention as they provided opportunities for the research partners to reflect collaboratively with particular groups of stakeholders in mind.

First, the research team developed a logic model (Table 2). The logic model delineated four key values at the foundation of the program, each targeting different stakeholder groups: patient autonomy (patients), interdisciplinarity (clinicians), equity (administrators, community organizers, and decision-makers), and quality assurance (researchers). Participants representing the different stakeholder groups were involved throughout the participatory research process to ensure that the program and/or knowledge developed through the research addressed these groups' specific needs. In the logic model, different outputs were conceived for each group, based on the needs they identified throughout the process. Through their collaborative involvement in the research, patient partners would develop recruitment material; clinicians would develop recruitment, training material, and implementation guides; administrators would provide resources to support the program's implementation and evaluation; researchers would help ensure the knowledge generation processes followed rigorous standards.

**Table 2:** Proposed model guiding the knowledge translation of the DEGP.

Rationale	To provide a health care service that will improve patients' control over their diabetes.		
Values	Patient autonomy	Interdisciplinarity	Health equity and quality assurance
Target audience	Patients	Clinicians	Administrators, community organizations, and other decision- makers
Primary objective	To offer a health care service that will improve patients' sense of empowerment in relation to their diabetes.		
Secondary objectives	To ensure that the program meets the needs of patients with diabetes and diabetes care providers	To ensure that patients, clinicians, and other stakeholders are involved in the development and implementation, and evaluation of the program	To ensure that the program is feasible, integrated, and cost effective in our health care system
Intervention	Participatory research on the Diabetes Empowerment Group Program and development of knowledge translation outputs		
Knowledge translation activities	Collaborative development of: DEGP content Recruitment material Empowerment assessment tool (MEA-D) Facilitation of DEGP sessions	Collaborative development of: DEGP content Training material Program evaluation (i.e., MEA-D, clinical outcomes) Facilitation of DEGP sessions	Participate in: Aligning program objectives with institutional and health policy objectives Program evaluation (i.e., definition of clinical outcomes, cost effectiveness, feasibility)
Resources	Patient partner and participatory research support Remuneration for patient partnership	Participatory research and quality improvement support Interdisciplinary partners Flexibility regarding schedules and care delivery	Identification of population needs to improve diabetes outcomes Identification of resources available to allocate toward diabetes care programs (funding for material and human resources) Administrative support for the DEGP Research support for program evaluation
Outputs	Recruitment strategies among peers Empowerment assessment tool (MEA-D) Social and civic mobilization of patient partners	Implementation guide Empowerment assessment tool (MEA-D) Evidence of clinical markers of diabetes control Enhanced culture of collaboration with patients and other stakeholders	Evidence of improved diabetes management and population engagement measures Evidence of satisfaction among patients and health care providers Enhanced culture of collaboration with patients, clinicians and researchers

Second, in support of the evaluation of the DEGP, the research team developed and validated the McGill Empowerment Assessment - Diabetes (20). This measure of empowerment related to diabetes self-care addresses the four domains of Christens' (19) empowerment framework: attitude, emotion, behavior, and relatedness. In their development of this assessment tool, the team was prompted to reflect on the various theoretical dimensions of empowerment and how the DEGP addresses them.

Third, the patient partners involved in the program and research project developed recruitment material targeting people living with diabetes from the community. Specifically, the patient partners worked with a graphic designer to develop a recruitment brochure. This activity deepened the research team's understanding of what the program means to people living with diabetes. The reflection informing this brochure led to the patient partners co-authoring a commentary on their participation in the DEGP and this research (9).

Finally, various aspects and stages of this research were presented at scientific conferences or described in scientific manuscripts. These helped tailor messages to clinicians and researchers. They drew out the lessons learned from having developed and implemented the DEGP, and having conducted collaborative, practice-based research involving researchers, clinicians, and patient partners.

### Considerations of Acceptability and Feasibility

Several key considerations emerged from the discussions regarding the acceptability and feasibility of implementing or adapting the DEGP (Table 3). Informed by Greenhalgh's Diffusion of Innovations framework (11), these considerations are categorized according to: characteristics of the DEGP, system characteristics, setting (health institution) characteristics, and adopter/participant (patient, clinician, and administrator) characteristics. These are elaborated below.

**Table 3:** Considerations for feasibility and acceptability of implementing the DEGP.

**Themes**	**Details**	**Considerations**
**Innovation characteristics**		
	Based on empowerment framework	Ability to develop activities that promote enhanced knowledge, skills, attitudes, and relatedness among patients.
	Aligns with best practice recommendations	Multidisciplinary services: are patient centered, promote self-care and are situated within the Patient Medical Home
**System characeristics**		
	System readiness for innovation	• Existence of similar programs and perceptions about the quality of services provided • Culture of program assessment, quality improvement, and innovation is fostered • Leadership, expertise, and change management support
	Sociopolitical context	• Health care user (patient) power and influence in health care services • Centralization vs. decentralized health care policy • Incentives and mandates align with DGEP objectives
	Knowledge translation	Diffusion and dissemination of experiences and new knowledge through institutional and primary care research networks
	Expectations regarding health professionals' roles	Flexibility regarding health professionals' (physicians, nurses, and other allied health professionals) domain of practice and remuneration
	Agility responding to populational needs	• Readiness and capacity of healthcare system to respond to populational needs and gaps in current diabetes services: • Decision making devolves to front line teams • Interventions conceived around populational needs • Strong primary care health care foundation and advocacy
**Setting characteristics**		
	Capacity to allocate material and human resources	• Flexibility regarding clinician schedules • Administrative support • Available Space for group meetings
	Culture of innovation	• Staff's training and experience with innovation and implementation • Identification and promotion of clinical change champions and early adopter of practice changes
	Multidisciplinary health professional team	• Promotion of patients' sense of belonging within Patient Medical Home • Links to community services and ability to collaborate and remunerate community professionals
**Adopters/participants**		
**Patients**
	Perceived value	• Dissatisfaction with existing services • Perceived value of group medical visits • Understanding of empowerment • Patient desire to actively engage in content and development of services offered • Perceived benefit of group visits
	Recruitment and retention	• Motivation to engage with others and commit to extended number of visits • Clear objectives of group program • Perceived benefit of participation in DGEP • Reaching orphan patients • Capacity to devote time
**Clinicians**		
	Perceived value	• Need for improvement of existing services • Understanding of empowerment • Perceived benefit of facilitator role and collaborative group dynamic
	Recruitment and retention	• Dedicated time, resources and adequate remuneration • Perception of patient benefit • Enjoyment of participation
**Administrators**		
	Perceived value	• Relative advantage over existing programs or ability to integrate with current services • Perceived value of patient engagement

#### Characteristics of the DEGP

One of the key factors facilitating DEGP's acceptability is its alignment with best practice recommendations. The program follows a multidisciplinary approach, as recommended by Diabetes Canada, and emphasizes the patients' role in self-care.

The program was also developed according to a relatively comprehensive empowerment framework, comprising four dimensions: knowledge, skills, attitudes, and relatedness. The relational aspect was highlighted as one of the major advantages of the DEGP over other offered diabetes care services.

I liked when you were talking about empowerment. To me, the biggest barriers are the guidelines. This is somebody else's rule… and I spend all the. I don't look at the actual person; I look at a whole bunch of numbers. And I think it's actually harmful. Like you say, the disease is theirs – or the reality is theirs, and their life is theirs, and they have to… This has to be part of their life, or they're not going to continue it. There has to be something in it for them. If they are going to tell you what are the costs of whatever you are asking or suggesting to them – I think they are more likely to do this in a group. (Family physician)

This program provides a dynamic space to learn and share from each other, where patients' priorities and concerns are taken into account.

It's so different than in individual appointments; and hearing of everybody's different perspective… And we had time. So these conversations didn't have to be blunted and really short. And I think probably some of those more complex ideas, about being an individual and having to manage your diabetes within your life that's different than someone else's life - there is just more space for it… (Nurse practitioner)

#### System Characteristics

One of the major perceived barriers to implementing the DEGP was the existence of government-mandated chronic care programs targeting persons with diabetes. The interview subjects expressed reservations about investing additional resources or adapting current resources to develop and implement innovations such as DGEP.

I think it would be a shame if we weren't able to take advantage of some of the great things that have come out of your program, but how to incorporate it into what exists already, in terms of a focus on what components of empowerment – how can we introduce that into some of the structure of the program we have already? How can we introduce that into the classes? How can we learn from that, how can we make the program better? (Continuous quality improvement Coordinator)

Governments differ according to their preference for centralized or decentralized decision-making structures. More centralized power tends to leave less room for deviation or innovation, and health services may, as a consequence, fall short of meeting the needs of particular populations (21, 22). The Quebec Ministry of Health had recently enacted changes to the governance structure of regional health authorities and family medicine groups' operations, which left clinicians feeling overwhelmed.

Right now there are so many changes, and people are a bit overwhelmed with everything. So getting something new right now—anything, even if it's a wonderful idea—I'm not sure how it will get received. The people, we know… in the media, everybody says… I could feel it from the people I worked with. It's like… “Not another thing, please!” - (Academic family medicine group Program Director)

Changes in legislation can shift priorities toward different activities, whereby health care providers and patients must negotiate new avenues for addressing their own priorities.

It's all about leadership. It's all about somebody committed, and in a leadership position, and getting people on board—which is the idea of it, but in our restricted circumstances, we also really have the practical issue of limitation of personnel at all levels. (Family physician)

Other important system-level factors reported by the participants that constrain innovation are remuneration and performance incentives and disincentives. In Quebec, physicians are self-employed and bill the provincial government for their services. Physicians are primarily remunerated *via* fee-for-service in primary care settings, with an additional sum based on client enrollment (23). In cases where physicians are concerned about meeting the client enrollment requirements, nurses and allied health professionals could play a more prominent role in delivering the program.

#### Setting Characteristics

Based on experience conducting the DEGP pilot, requisite material, and human resources to run the program were identified. Space, where the group meetings are to be held, is needed. The program also demands some administrative work, such as: contacting patients who had expressed interest in participating, identifying availability, and establishing a schedule. In addition, flexibility regarding clinicians' schedules may be needed to work around the interested patients' availability (possibly in the evenings and weekends).

At our [family medicine group] I think it would be very feasible to implement the program, because we already have a nutritionist who just started; we have 5 new nurses, I think their schedules are still open. We're open on Saturdays and Sundays. So if we were to want to have the meetings on weekends, we would have a space upstairs. (Nurse practitioner)

The staff's training and experience innovating and successfully implementing programs were also considered major assets. Change management is considered an important factor in successful implementation in health care settings (24, 25). In settings that foster a culture of innovation and continuous quality improvement, the staff has cultivated knowledge and skills to evaluate needs, mobilize resources, and modify existing practices.

What we're trying to do with [continuous quality improvement] right now, is to try to make our decisions about what we—where we focus our efforts, where we focus our resources and time is based on solid data and evidence for the efficacy of whatever it is we're going to work toward. So that's what we're trying to shift toward right now – as a philosophy for the entire clinic, as a strategic approach to how we use our resources. So I could tell you that would weigh very heavily in how we try to decide how we move forward with any type of initiative right now. I don't think that was the case before; but it's certainly something we are trying to move toward now. (Continuous quality improvement coordinator)

Finally, at the practice level, another asset to DEGP implementation is its alignment with the Patient Medical Home model, which emphasizes multidisciplinary care and links to community services. Settings that embrace multidisciplinary care have a more developed and refined practice of inter-professional collaboration.

#### Patients

The DEGP requires a sufficient number of people living with diabetes who are willing and able to commit 2 h of their time, on occasion, to group visits. Attracting potential participants implies that they perceive some value in what the program offers, such as engagement with others living with diabetes or playing a greater role in determining the discussions' content.

There are definitely people who are afraid of group settings. They only want their doctor, it's a private matter; “No one sees my file, and I'm not going to start talking to anybody else about whatever problems I have.” (Patient partner)

It also implies that they understand their condition as something requiring some attention.

The young pre-diabetic, they are not very interested… Very often they don't even want to see a dietician, or a kinesiologist; and the dropout rate is quite high. Because they don't feel… I mean they're young… They don't feel sick; they say, “Well, why should I come and spend time? I know what to do.” But they just don't do it. So I think it might be a barrier to recruit these people into groups. For those who are on active treatment, I think maybe it's a different story; they feel they are sicker, because they have to take pills. But with the pre-diabetics especially, we have a hard time. (Family physician)

It was suggested that clinicians could target more homogeneous groups, including those who would not typically attend standard cardiometabolic prevention programs, patients with lower literacy levels, specific cultural or marginalized groups, or uncontrolled or newly diagnosed patients.

I'm wondering too if in your groups you had people from similar cultures; because even things like food, right, it's so cultural. You have people who eat rice for each of their meals, and large amounts. I find it hard – I'm not going to tell someone to cut out… I mean, I know it's a lot of the dietician's role as well, but still; food is a huge factor, so if patients were able to help each other. (Nurse Practitioner)

The patient partners suggested that recruitment may be more successful if they receive an invitation from their primary health care professional directly and get the opportunity to discuss the potential benefits of this kind of program. Scheduling was considered an important challenge by the participants, which could limit certain populations' capacity to attend depending on the time selected.

We definitely had more retired patients; we did do an evening as well to try to accommodate; but it was true that even though we made the evening accommodating, it was the more difficult one, still. (DEGP Nurse)

Another major concern we had with the cardio-metabolic program when I was involved with it was getting your younger working diabetic to come to so many meetings. Because many of them have a young family; they work full-time, they work downtown. There is no parking here. And it was a huge barrier. It really was. We tried evening classes, we… We really tried various things; and they were like, “But it's too late, I'm tired at the end of a work day.” (Family physician)

Retention of participants was also considered an important element of implementation planning. The high retention rate of the DEGP seemed, to the discussants, to suggest that the relational and patient-centered focus of the DEGP and the fixed-cohort structure may have improved patients' commitment to the program.

After the first meeting, I was hooked. I think my neighbor was too. The idea that you're not there in a… lecturing sense, listening to someone go on. You're actually participating, or you're talking. Or somebody would say, “Hey I've got that problem too, and this is how I dealt with it.” And it's more… you're drawn into it more, and you learn more. And there's more… buy-in, if you like, in participating, rather than sitting there and being lectured to or being instructed. (Patient partner)

The patients' perceived benefit of their participation in the program reportedly motivated their desire to return and continue to engage in self-care. One of the patient partners explained that their interactions with others in the DEGP made a profound, lasting impression on them: “a good group stays with you for life.”

Furthermore, to ensure better retention, it was suggested that the facilitators provide the participants with clear objectives and regularly solicit the participants for feedback, as was done in the pilot program.

#### Health Professionals

Considerations were raised regarding the attraction of facilitators and allied health professionals to the program. The DEGP physician and nurse were initially interested in developing the DEGP to address shortcomings of existing services and create a fun, engaging, and collaborative space for health care professionals and patients to discuss living with diabetes, the challenges people face, and the strategies they have put in practice. This collaborative, multidisciplinary, patient-centered approach appeared to appeal to the health professionals who participated in the group interviews.

I think the concept is wonderful; and I think a lot of patients that we see, as it was said, it's as if we're lecturing them. And most of the time I find myself saying, “I don't want to be lecturing you when I'm saying those things, but it's really important.” It might be just things that I'm saying, and they're like, “Okay, okay, I've heard this a million times before.” But the fact that they're in a room with other people sharing the same disease and condition! (Pharmacist)

As with the patients, the health professionals' perceived benefit (including the perceived need to improve existing services) and their enjoyment of participation would increase their likelihood of participating in subsequent sessions and/or groups. As the visits were longer and more frequent than individual visits, the clinician-patient therapeutic alliance was more quickly and easily developed. In addition, the close collaboration with fellow health professionals further increased their satisfaction. The patient-centered agenda and group dynamic brought about topics that were more pertinent and meaningful for patients. The clinicians noted that the topics brought up by patients were some that they might never have considered discussing (e.g., shame in carrying a diabetes diagnosis, perception of physician attitudes, cultural barriers, or political views on diabetes care). The clinicians who participated in the pilot reported that they found that the open interaction made the visits more gratifying.

Potential barriers reported by clinicians included the availability of adequate resources, the capacity to dedicate time and remuneration. Support of an administrative coordinator is recommended to assist with the program's coordination, including recruitment, contacting patients, and group visit logistics. Regarding time, the DEGP was considered more feasible in settings where clinicians had time and flexibility in their practices to take part in programs such as this. Working in clinics with experience in championing innovations or a stronger culture of innovation and partnership with patients was considered an asset. Physician remuneration may represent a challenge in some settings. However, where physicians cannot apply a group billing code and are remunerated according to a fee-for-service model, they may bill for individual medical consultations since the meeting consists of a formal medical visit. Group facilitation, however, need not necessarily be conducted by a physician. As mentioned above, where physicians' remuneration is more challenging, nurses and allied health professionals could play a more prominent role.

#### Administrators

To support the implementation of the DEGP, it was suggested that administrators be provided evidence that this program is more effective than existing programs.

They [administrators] are very big on indicators. “Show us why this is a good program to adopt.” (Nurse manager)

Developers of novel interventions often face the challenge of needing to provide evidence of effectiveness, with limited capacity to conduct pilot studies. Consequently, especially when budgets are restricted, many new programs are developed top-down rather than bottom-up.

Interview facilitator to interview subjects: “From your experience at your individual sites, what was helpful in starting new initiatives? […] What were some of the elements that actually made things happen?”

Continuous quality improvement coordinator: “Well, top-down mandates…”

Family Physician: “Being dictated from the top! Literally being told: this is what we're doing.”

The initial, small-scale pilot's primary aim was to assess the feasibility of DEGP. Given the pilot's scale, causal claims about its cost-savings or its improvement of diabetes or self-care outcomes could not be established. In order to demonstrate the effectiveness of the DEGP, a larger, sufficiently-powered pilot will need to be conducted. While data on its effectiveness is unavailable, the participants suggested that attracting select administrators may be achieved by highlighting that perceptions of the program are favorable and that it is considered an improvement over existing interventions, in concept, since the DEGP was conceived to address some of their shortcomings. For instance, the pilot's 90% retention rate suggests that the program may be more attractive to patients than the current government-mandated Cardiometabolic Program in Quebec, which was found to have a retention rate of <60% (26).

## Discussion

### Trustworthiness

Regarding the trustworthiness of the results generated through this research, data sources and participatory processes speak to the credibility of findings (27, 28). The description of the DEGP was informed by discussions with the healthcare providers and patients involved in the pilot. In addition, the interview guide was collaboratively developed by the participatory research team, which includes researchers, clinicians, and patient partners. The triangulation of data further contributed to the credibility of the findings. Discussions with patients and healthcare providers from other settings enabled a deeper understanding of the program and a description that encompasses diverse perspectives and addresses different understandings.

Another key consideration is the transferability of the findings to other settings (28). We described the major themes regarding the feasibility and acceptability of implementing the DEGP that emerged from discussions with patient partners and healthcare professionals from three different settings in Montreal. As above, the triangulation of data from interviews with the DEGP team and healthcare providers from other settings helped ensure a more comprehensive description of the program, which incorporates a variety of healthcare providers' perspectives. Therefore, we expect that the present description of DEGP might inform the implementation of this program or its adaptation in Quebec and internationally.

### Future Directions

The DEGP is a novel, patient-centered program for people living with diabetes that emphasizes multidisciplinary collaboration, group dynamics, and relational aspects of self-care. We provided a detailed description of the DEGP and identified key factors pertaining to the feasibility and acceptability of its implementation or adaptation in other healthcare settings. We hope that the present research drives improvements to current programs and the development of the DEGP in other settings. To further support its implementation and adaptation, we are currently developing an implementation guide, which will provide more concrete and practical recommendations. We also developed and validated a measure of empowerment (MEA-D McGill Empowerment Assessment -Diabetes) (20), which will better enable an assessment of participants' needs prior to the program, and measurement of change following the program. We plan to disseminate these documents and tools across Quebec and Canadian research networks.

Further pilot research is needed, using a larger cohort, in order to demonstrate the effectiveness of the DEGP in terms of health outcomes, cost savings, and validated measures of empowerment. Once the DEGP is implemented or adapted in other healthcare settings, we also plan to explore further the usefulness of the conceptual table (Table 3), for clinical practice stakeholders, in addressing the feasibility and acceptability of implementing the DEGP or other group-based primary care practice innovations. Additional developments of this program may include: involvement of patient partners in facilitation, development, and evaluation; evaluating the feasibility of DEGP implementation in diverse settings and populations; and the inclusion of psychosocial outcomes, as is increasingly prominent in evaluating the effectiveness of diabetes programs.

## Conclusion

The DEGP is an innovative, patient-centered medical care program conceived to educate and motivate diabetes self-care in a collaborative environment. It responds to recommendations from Diabetes Canada for a multidisciplinary approach in support of patients' management of their diabetes. It addresses some of the shortcomings of existing programs by emphasizing the relational impact of group activities and patient engagement in the program content, following a collaborative, multidisciplinary framework. Patients expressed appreciation for the opportunity to share and learn with patients and health care providers alike. Clinicians expressed interest in integrating elements of the DEGP into existing diabetes self-care programs, if provided adequate resources. Administrators appeared favorable toward this novel program, in concept, but explained the challenge of allocating material and human resources without sufficient evidence, and expressed reticence in supporting programs that lie outside ministerial or institutional priorities. The participatory research process broadened the participants' understanding of the DEGP and the needs of different stakeholders. This research provides a detailed description of the DEGP and insight on the factors that may impact the feasibility and acceptability of implementing similar group-based primary care interventions in other settings.

## Data Availability Statement

The datasets presented in this article are not readily available because The institutional review board has not granted permission to share this data (interview transcripts). Requests to access the datasets should be directed to Fanny Hersson-Edery, fanny.hersson-edery@mcgill.ca.

## Ethics Statement

The studies involving human participants were reviewed and approved by McGill University Faculty of Medicine Institutional Review Board. The patients/participants provided their written informed consent to participate in this study.

## Author Contributions

FH-E prepared the manuscript and all authors revised and approved the final version. All authors contributed to the conception and design of the study as well as data collection, analysis, and interpretation of results.

## Conflict of Interest

The authors declare that the research was conducted in the absence of any commercial or financial relationships that could be construed as a potential conflict of interest.
